# ‘Stop going off on a tangent’: a novel method for discriminating pathological from tangential fluorescence during photodynamic diagnosis cystoscopy

**DOI:** 10.1308/003588412X13373405387096m

**Published:** 2012-05

**Authors:** R Nair, C Coker

**Affiliations:** ^1^London Deanery,UK; ^2^Brighton and Sussex University Hospitals NHS Trust,UK

## BACKGROUND

Fluorescence photodynamic diagnosis (PDD) cystoscopy is gaining credence in the diagnosis and surveillance of bladder cancer. It combines normal white light cystoscopy with periodic blue light examination of the bladder pre-instilled with a tumour selective porphyrin, hexa-aminolevulinic acid (hexa-ALA).^1^ This method detects areas of fluorescent tissue suggestive of tumour or carcinoma in situ, and has been shown to decrease recurrence rates and improve outcomes when compared with white light cystoscopy.^2^

The mucosa of the bladder neck and lateral bladder wall is viewed at an angle on blue light cystoscopy and displays tangential fluorescence following hexa-ALA instillation. Although a useful observation, by acting as a positive control,^3^ a common conundrum remains the discrimination between pathological and tangential fluorescence. We describe a simple technique to aid in discriminating between the two.

## TECHNIQUE

**Figure 1:**
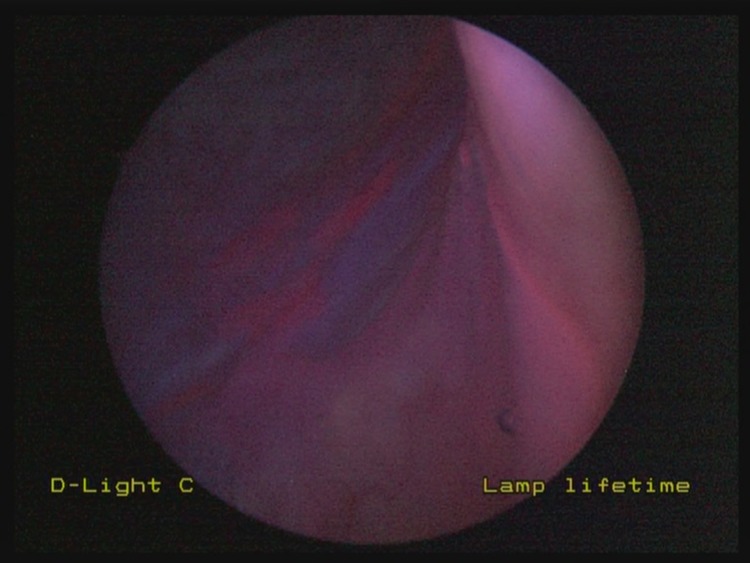
An area of fluorescence identified on the right lateral bladder wall on blue light cystoscopy. This area of fluorescence may represent tangential or pathological change.

**Figure 2:**
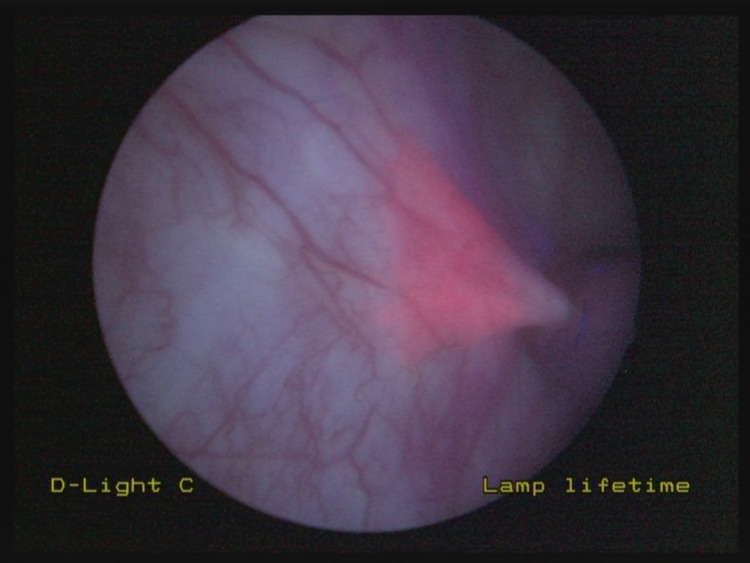
On elevation of mucosa adjacent to the area of concern with endoscopic cold cup biopsy forceps, this area of non-specific fluorescence disappears, suggestive of tangential change.

**Figure 3:**
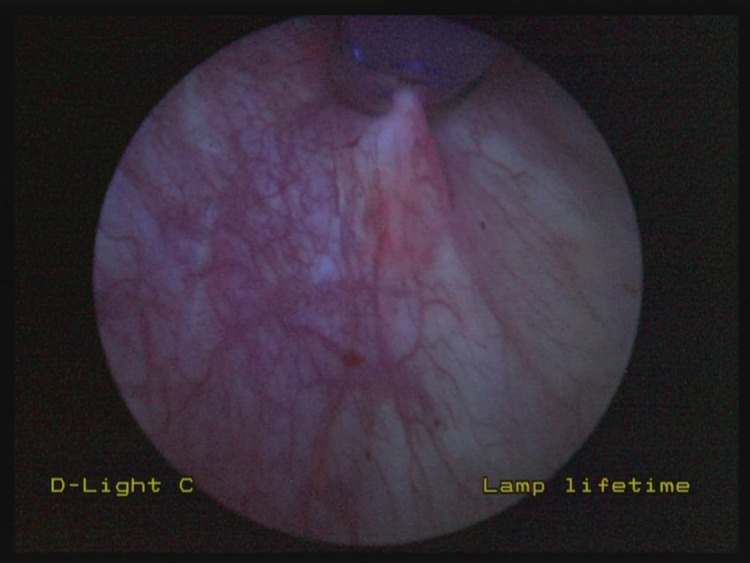
In this situation, with elevation of adjacent mucosa with endoscopic cold cup biopsy forceps, the area of non-specific fluorescence remains. This may represent pathological fluorescence and representative biopsies were taken confirming this.

When an area of non-specific fluorescence is encountered on blue light cystoscopy (Fig 1), endoscopic cold cup biopsy forceps are used to raise gently the mucosa adjacent to the area of concern. This allows for tenting of the suspicious area, altering the angle at which the blue light is placed on this region. If the area of non-specific fluorescence disappears, it is likely to represent tangential fluorescence (Fig 2). If, however, the region in question remains fluorescent despite tenting and manipulation, it may represent pathological fluorescence and representative biopsies should be taken (Fig 3).

## DISCUSSION

We recommend this technique as a method for discriminating between pathological and tangential fluorescence during PDD cystoscopy. It allows for targeted intervention and avoids unnecessary biopsies.
